# Supplementary material for: Aging is associated with functional and molecular changes in distinct hematopoietic stem cell subsets
Source: Nat Commun. 2024 Sep 11;15:7966. doi: 10.1038/s41467-024-52318-1 (PMC11391069; doi:10.1038/s41467-024-52318-1)
Supplement: Supplementary file 1 — Supplementary Information [file 41467_2024_52318_MOESM1_ESM.zip › Supplementary Information_240909.pdf]

Supplementary Fig. 1, related to Figure 1

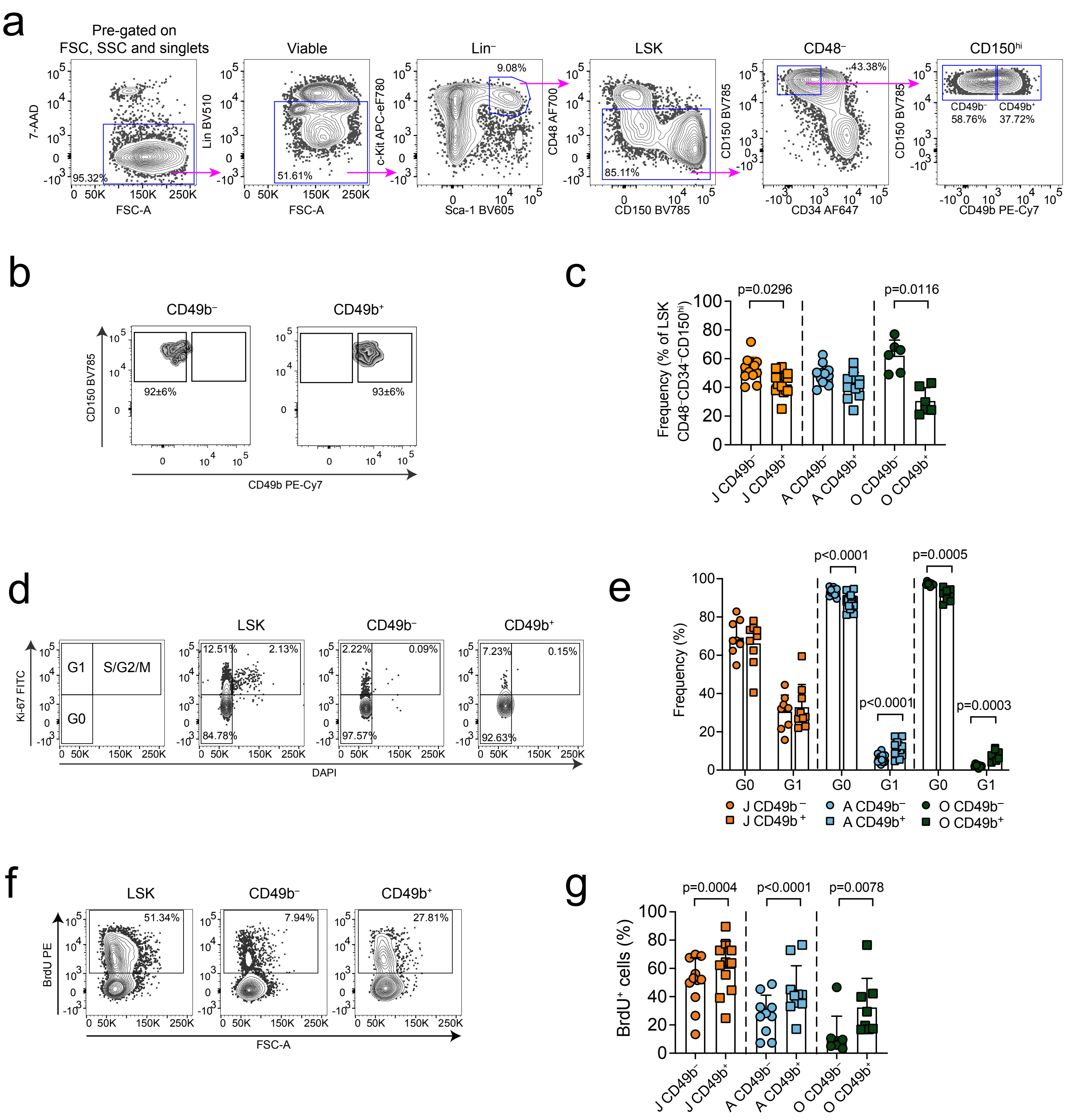

Supplementary Fig. 1, related to Figure 1

Fluorescence-activated cell sorting and functional evaluation of CD49b$^-$ and CD49b$^+$ HSC subsets in aging. **a**, Representative FACS profile and gating strategy of the phenotypic HSC compartment (Lin$^-$Sca-1$^+$c-Kit$^+$ (LSK) CD48$^-$CD34$^-$CD150^{hi}) and further separation with CD49b. Frequency of parent gates are shown. **b,** Sort purity analysis of CD49b$^-$ and CD49b$^+$ populations pre-gated on Lin$^-$Sca-1$^+$c-Kit$^+$CD48$^-$CD34$^-$CD150^{hi}. Sort purity is represented as mean ± s.d. for each subset, from 34 experiments. **c,** Frequency of CD49b$^-$ and CD49b$^+$ HSC subsets within the LSK CD48$^-$CD34$^-$CD150^{hi} population in juvenile (n=13 mice, 3 experiments), adult (n=12 mice, 6 experiments), and old (n=6 mice, 6 experiments) mice. **d,** Representative FACS profile of Ki-67 cell cycle analysis. **e,** Frequency of CD49b$^-$ and CD49b$^+$ HSCs in G0 and G1 of juvenile (n=9 mice, 3 experiments), adult (n=15 mice, 6 experiments), and old (n=8 mice, 5 experiments) mice. **f,** Representative FACS profile of BrdU cell proliferation analysis. **g,** Frequency of BrdU$^+$ CD49b$^-$ and CD49b$^+$ HSCs of juvenile (n=11 mice, 3 experiments), adult (n=10 mice, 3 experiments), and old (n=8 mice, 3 experiments) mice.

Mean ± s.d. is shown. The statistical analysis was performed with paired t-test except juvenile G1 in (e) and the old age group in (g), where the Wilcoxon signed-rank test was used. J, juvenile; A, adult; O, old.

Source data are provided as a Source Data file.

Supplementary Fig. 2, related to Figure 2

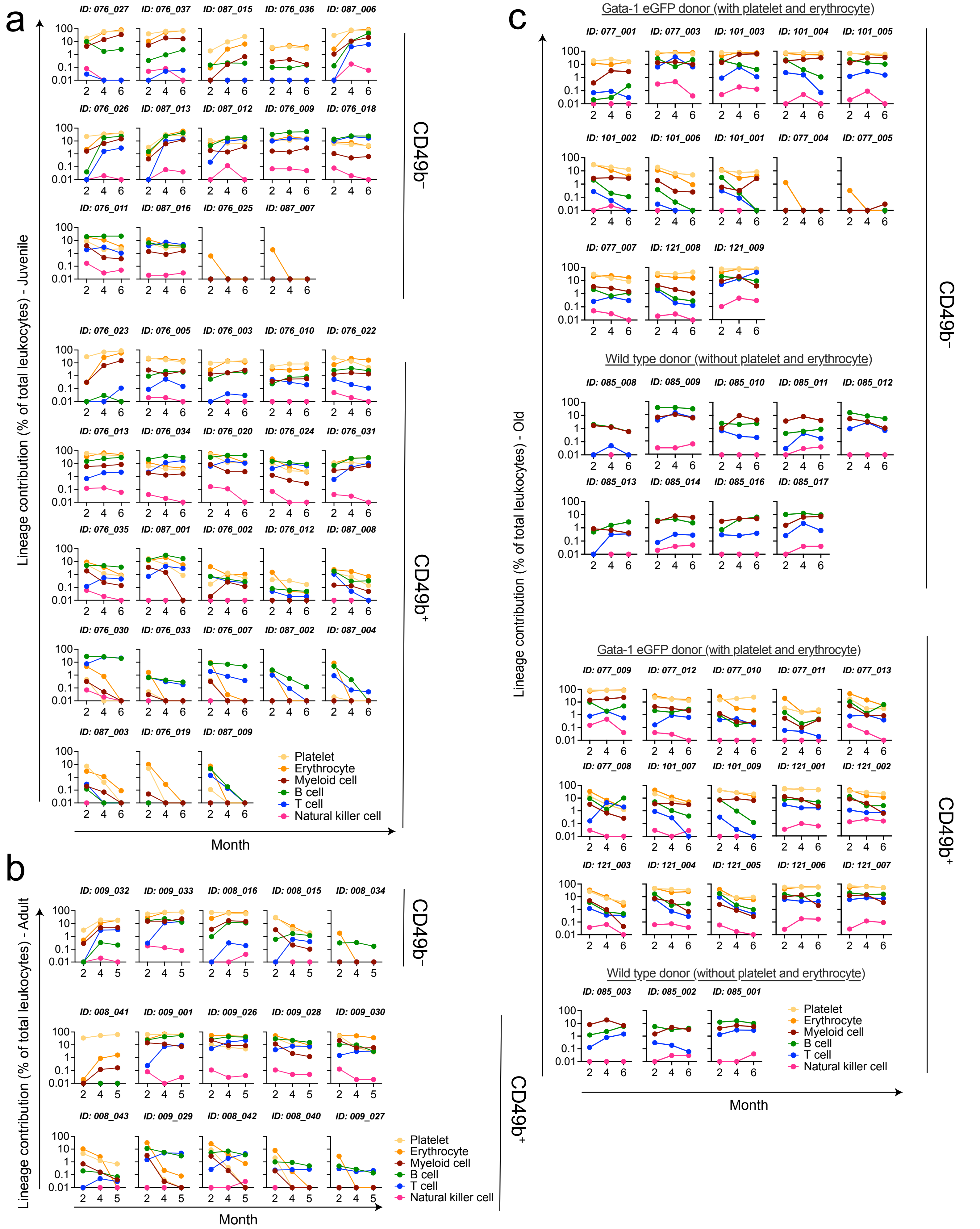

Supplementary Fig. 2, related to Figure 2

Repopulation profiles of primary transplanted mice from juvenile, adult, and old HSC subsets. **a,** Total donor-derived blood lineage contribution in the peripheral blood of individual mice transplanted with $CD49b^{-}$ and $CD49b^{+}$ HSC subsets from juvenile mice in primary transplantation ($n_{CD49b^{-}}$=14 mice and $n_{CD49b^{+}}$=23 mice, 2 experiments). **b,** Total donor-derived blood lineage contribution in the peripheral blood of individual mice transplanted with $CD49b^{-}$ and $CD49b^{+}$ HSC subsets from adult mice in primary transplantation ($n_{CD49b^{-}}$=5 mice and $n_{CD49b^{+}}$=10 mice, 2 experiments). **c,** Total donor-derived blood lineage contribution in the peripheral blood of individual mice transplanted with $CD49b^{-}$ and $CD49b^{+}$ HSC subsets from old mice in primary transplantation ($n_{CD49b^{-}}$=22 mice and $n_{CD49b^{+}}$=18 mice, 4 experiments). Transplanted mice are grouped according to transplantation with wildtype C57BL/6J or Gata-1 eGFP donor mice.

Source data are provided as a Source Data file.

Supplementary Fig. 3, related to Figure 2

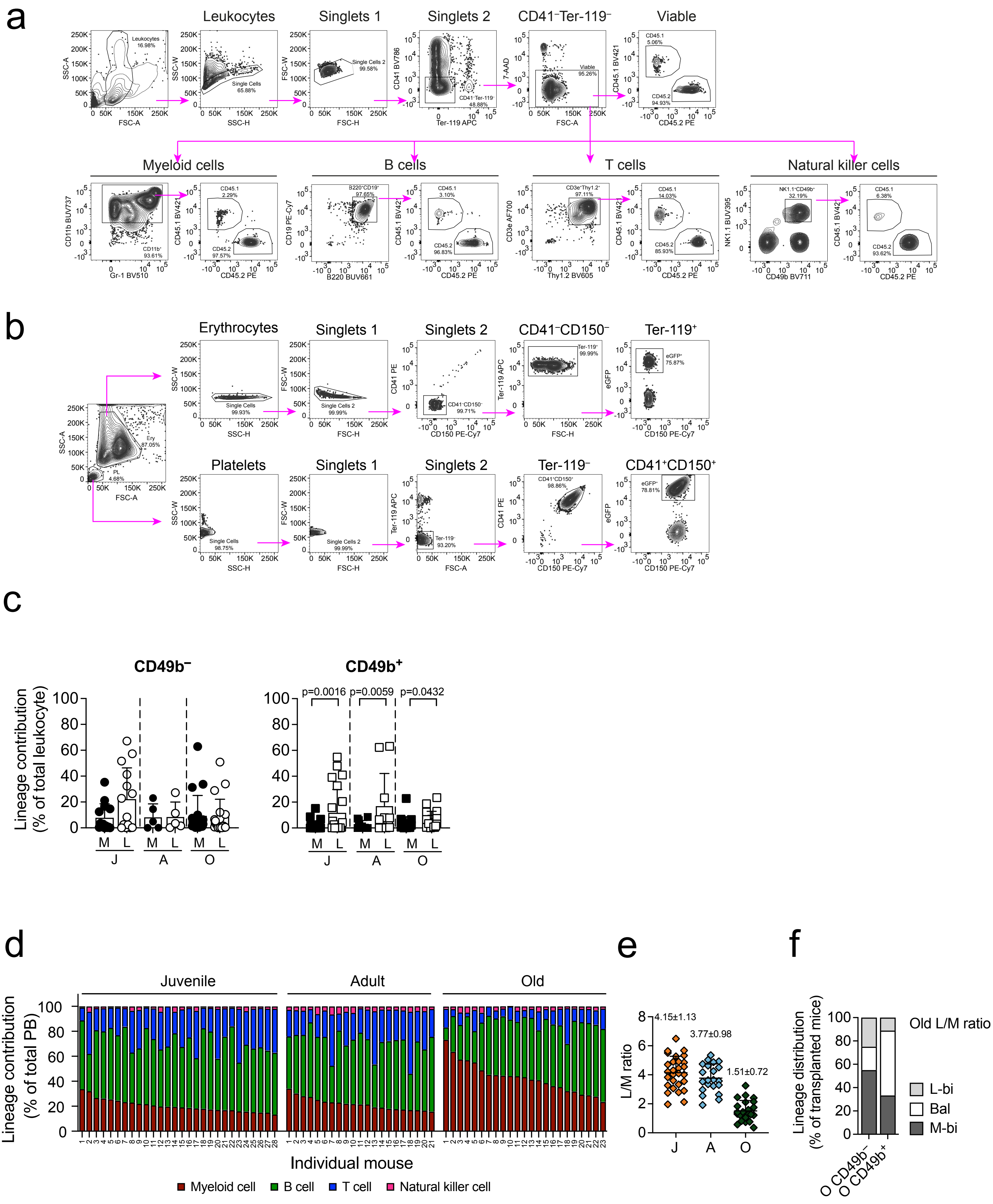

Supplementary Fig. 3, related to Figure 2

Flow cytometry analysis of mature blood lineages and blood lineage profiles in unmanipulated mice. a, Representative FACS profiles and gating strategies of donor-derived myeloid cells, B cells, T cells, and natural killer cells in the peripheral blood. Frequency of parent gates are shown. **b,** Representative FACS profiles and gating strategies of donor-derived platelets and erythrocytes in the peripheral blood. Frequency of parent gates are shown. **c**, Total lineage contribution to myeloid (M) and lymphoid cells (L: B, T, and NK cells) in the peripheral blood of mice transplanted with juvenile, adult, and old $CD49b^{-}$ and $CD49b^{+}$ HSC subsets, 5-6 months post-transplantation ($n^{J}_{CD49b^{-}}$=14 mice, $n^{J}_{CD49b^{+}}$=23 mice, $n^{A}_{CD49b^{-}}$=5 mice, $n^{A}_{CD49b^{+}}$=10 mice, $n^{O}_{CD49b^{-}}$=20 mice, and $n^{O}_{CD49b^{+}}$=18 mice). **d,** Relative contribution of myeloid, B, T, and natural killer cells in peripheral blood leukocytes of individual unmanipulated juvenile (n=28 mice, 3 experiments), adult (n=21 mice, 5 experiments), and old (n=23 mice, 3 experiments) mice. **e,** Calculated lymphoid (L: B, T, and NK cells) to myeloid (M) cell ratio in the peripheral blood based on unmanipulated juvenile, adult, and old mice from (d). **f,** Frequency of observed lineage distribution patterns of mice transplanted with $CD49b^{-}$ and $CD49b^{+}$ HSCs from old mice, 5-6 months post-transplantation, calculated using lymphoid/myeloid (L/M) cell ratio from old unmanipulated mice as a reference ($O_{CD49b^{-}}$=20 mice, $O_{CD49b^{+}}$=18 mice).

Mean ± s.d. is shown in (c,e). The statistical analysis was performed with paired t-test in $J_{CD49b^{-}}$, $A_{CD49b^{-}}$, and $O_{CD49b^{+}}$; while the Wilcoxon signed-rank test was used for $J_{CD49b^{+}}$**,** $A_{CD49b^{+}}$, and $O_{CD49b^{-}}$ in (c). J, juvenile; A, adult; O, old; M, myeloid; L, lymphoid; PB, peripheral blood; L/M, lymphoid to myeloid; L-bi, lymphoid-biased; Bal, balanced; M-bi, myeloid-biased.

Source data are provided as a Source Data file.

Supplementary Fig. 4, related to Figures 2-3

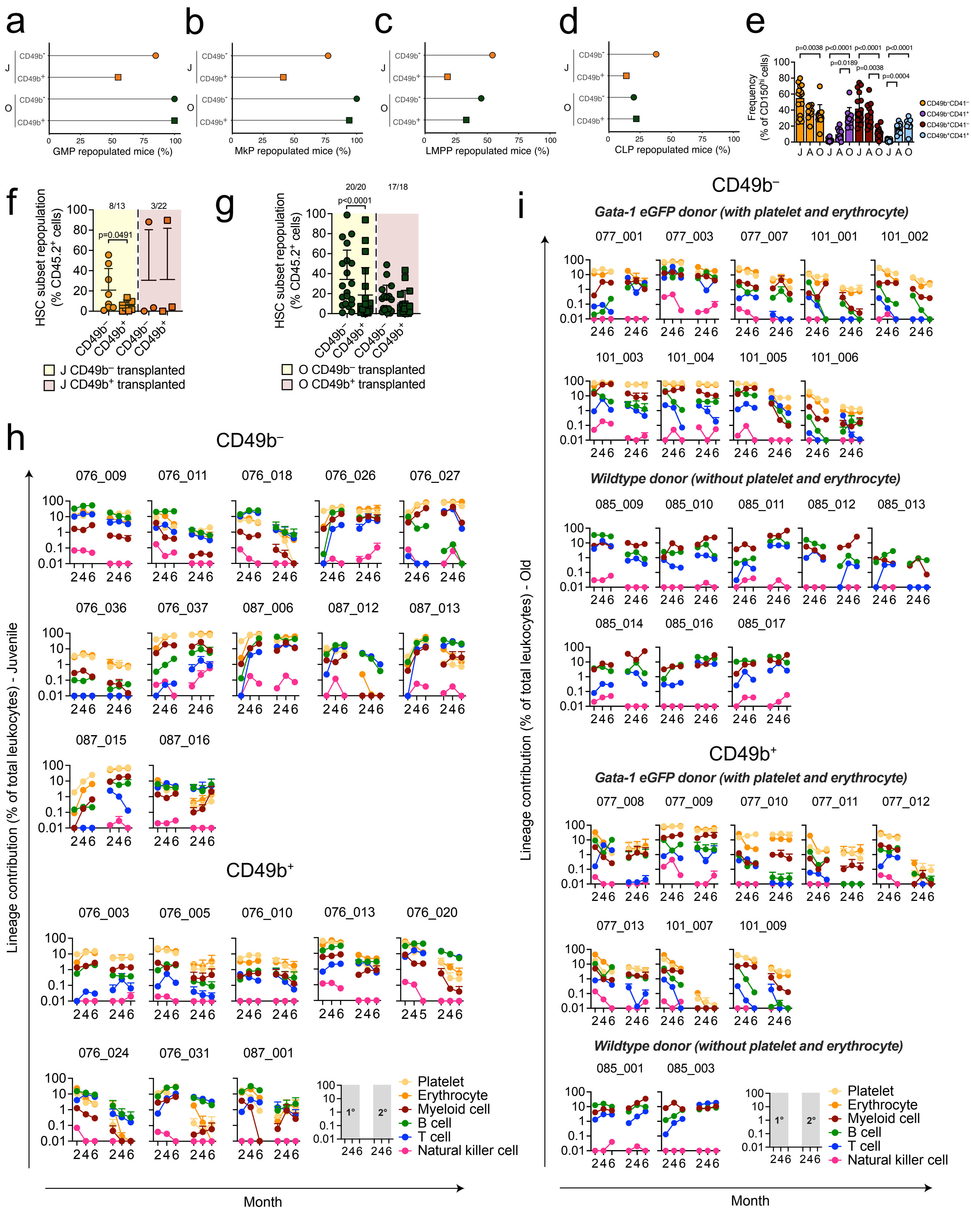

Supplementary Fig. 4, related to Figures 2-3

Repopulation of stem- and progenitor cell populations and secondary transplanted mice from juvenile and old HSC subsets. **a-d**, Proportion of mice with GMP, MkP, LMPP, and CLP repopulation in the bone marrow (BM), 6 months after primary transplantation of juvenile or old $CD49b^{-}$ and $CD49b^{+}$ HSCs. **e**, Frequency of $CD49b^{-}CD41^{-}$, $CD49b^{-}CD41^{+}$, $CD49b^{+}CD41^{-}$, and $CD49b^{+}CD41^{+}$ subsets within the phenotypic HSC compartment of juvenile (n=17 mice, 6 experiments), adult (n=11 mice, 7 experiments), and old (n=12 mice, 3 experiments) mice. **f,** Frequency of phenotypic $CD49b^{-}$ and $CD49b^{+}$ HSC repopulation in the BM of reconstituted mice transplanted with juvenile $CD49b^{-}$ and $CD49b^{+}$ HSC subsets 6 months post primary transplantation. **g,** Frequency of phenotypic $CD49b^{-}$ and $CD49b^{+}$ HSC repopulation in the BM of reconstituted mice transplanted with old $CD49b^{-}$ and $CD49b^{+}$ HSC subsets 6 months post primary transplantation. **h,** Total donor-derived blood lineage contribution in the peripheral blood (PB) of individual mice transplanted with $CD49b^{-}$ and $CD49b^{+}$ HSC subsets from juvenile mice in primary and secondary transplantations ($n^{J}_{CD49b^{-}}$=12 mice and $n^{J}_{CD49b^{+}}$=8 mice, 2 experiments, each primary donor mouse transplanted into 2-3 secondary recipients). **i,** Total donor-derived blood lineage contribution in the PB of individual mice transplanted with $CD49b^{-}$ and $CD49b^{+}$ HSC subsets from old mice in primary and secondary transplantations ($n^{O}_{CD49b^{-}}$=17 mice and $n^{O}_{CD49b^{+}}$=10 mice, 3 experiments, each primary donor mouse transplanted into 1-5 secondary recipients).

Mean $\pm$ s.d. is shown in (e-g). Kruskal-Wallis with Dunn's multiple comparison test was performed in (e), except for $CD49b^{+}CD41^{-}$, where one-way ANOVA with Tukey's multiple comparison test was performed. In (f,g), the number of reconstituted primary donor mice out of all analyzed mice is indicated above the graphs, and statistical analysis was performed with the paired t-test in juvenile and the Wilcoxon signed-rank test in old. In (a-d, f-g): $n^{J}_{CD49b^{-}}$=13 mice, $n^{J}_{CD49b^{+}}$=22 mice, $n^{O}_{CD49b^{-}}$=20 mice, $n^{O}_{CD49b^{+}}$=18 mice. J, juvenile; A, adult; O, old; GMP, granulocyte-monocyte progenitor; MkP, megakaryocyte progenitor; LMPP, lymphoid-primed multipotent progenitor; CLP, common lymphoid progenitor.

Source data are provided as a Source Data file.

Supplementary Fig. 5

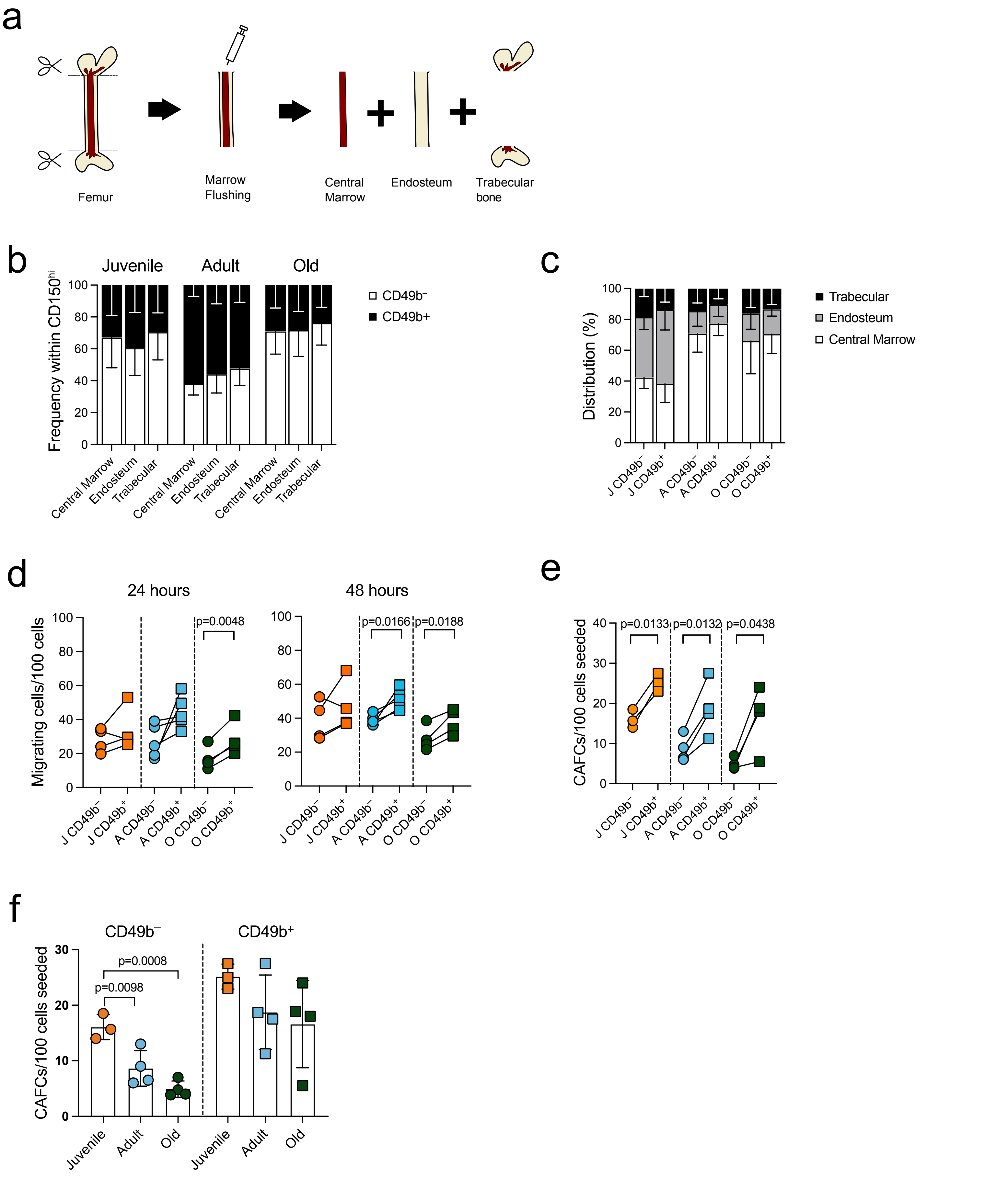

Supplementary Fig. 5

Distribution of CD49b^{-} and CD49b^{+} subsets in the BM and analysis of migration properties

a, Schematic overview of the separation of central marrow, endosteum, and the trabecular region of the femur. **b,** Frequency of CD49b^{-} and CD49b^{+} HSC subsets in the central marrow, endosteum, and the trabecular region of the femur in juvenile (n=9 mice, 4 experiments), adult (n=10 mice, 4 experiments), and old (n=4 mice, 4 experiments) mice. **c,** Distribution of CD49b^{-} and CD49b^{+} subsets in the central marrow, endosteum, and the trabecular bone of juvenile (n=9 mice, 4 experiments), adult (n=10 mice, 4 experiments), and old (n=4 mice, 4 experiments) mice. **d,** Number of the HSCs transmigrated across BM MSCs 24- and 48-hours post-seeding. HSC subsets from juvenile (n=4 mice, 4 experiments), adult (n=5 mice, 5 experiments), and old (n=4 mice, 4 experiments) mice were seeded. Migrated HSCs were calculated based on the numbers of non-migrated cells on top of the MSCs. **e,** Number of CAFCs formed from HSCs that have adhered and migrated through the MSC layer 28 days post-sort from juvenile (n=3 mice, 3 experiments), adult (n=4 mice, 4 experiments), and old (n=4 mice, 4 experiments) mice. **f**, Number of Cobblestone-Area-Forming Cell (CAFC) colonies formed in CD49b^{-} and CD49b^{+} HSC subsets 28 days post-sort.

Mean ± s.d. is shown in (b,c,f). Paired parametric t-test was performed in (d,e) except the 24 hour Juvenile age group, where nonparametric Wilcoxon signed-rank test was performed. One-way ANOVA with Tukey's multiple comparison test was performed in (f). J, juvenile; A, adult; O, old; CAFC, cobblestone area-forming cells.

Source data are provided as a Source Data file.

Supplementary Fig. 6, related to Figure 4

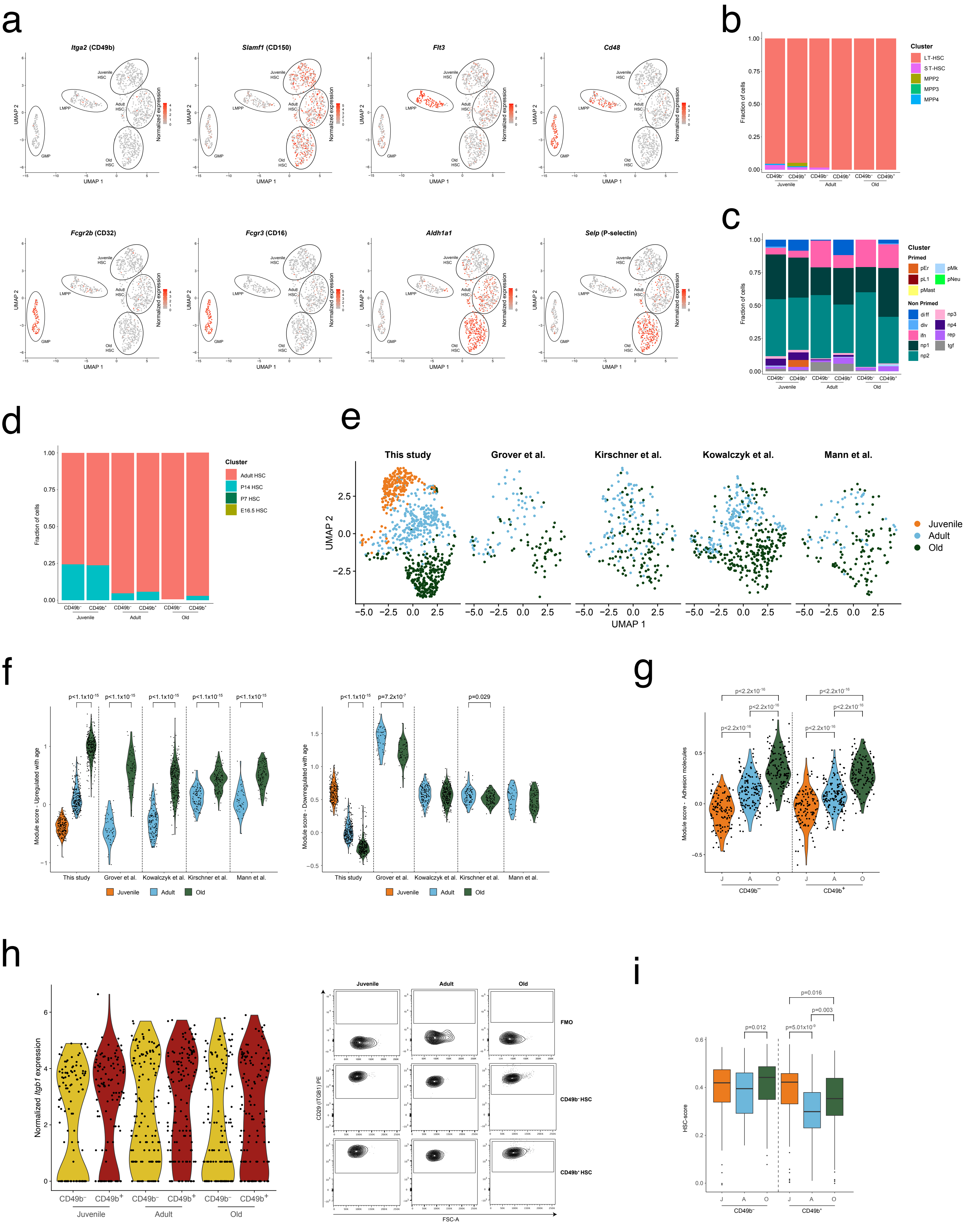

Supplementary Fig. 6, related to Figure 4

Gene expression of selected genes and contextualization of scRNA-seq data in relation to prior studies. **a**, UMAP visualization of gene expression of selected, characteristic cell surface markers and aging-associated genes by scRNA-seq. The color intensity represents normalized expression level. **b,** Distribution of HSC annotation to indicated cell types as defined by FACS in Rodriguez-Fraticelli et al.[32]. **c,** Distribution of HSC annotation to indicated expression signatures as defined by data from Hérault et al.[33]. **d,** Distribution of HSC annotation to HSCs from mice of indicated age as defined by data from Li et al.[34]. **e,** UMAP of integrated scRNA-seq data from this study (juvenile, adult, and old HSCs) and published datasets of adult and old HSCs. Each study is displayed separately. **f,** Module scores for gene sets with significant upregulation (left) or downregulation (right) with age in this study for the datasets included in (e). See Supplementary Data 1 for gene list. **g,** Module scores for cell adhesion molecules in juvenile, adult, and old HSC subsets. **h**, Expression of *Itgb1* in juvenile, adult, and old HSC subsets (left) and representative FACS profile of CD29 (ITGB1) expression in $CD49b^{-}$ and $CD49b^{+}$ HSCs from juvenile (n=9 mice, 4 experiments), adult (n=7 mice, 2 experiments), and old (n=3 mice, 2 experiments) mice (right). **i,** Calculated HSC-score for $CD49b^{-}$ and $CD49b^{+}$ HSCs from juvenile, adult, and old mice.

The statistical analysis was performed with Mann-Whitney test with adjustment for multiple comparisons in (f), One-way ANOVA with Tukey’s multiple comparison test in (g), and Kruskal-Wallis with Dunn’s multiple comparison test in (i). J, juvenile; A, adult; O, old.

Source data are provided as a Source Data file.

Supplementary Fig. 7, related to Figure 5

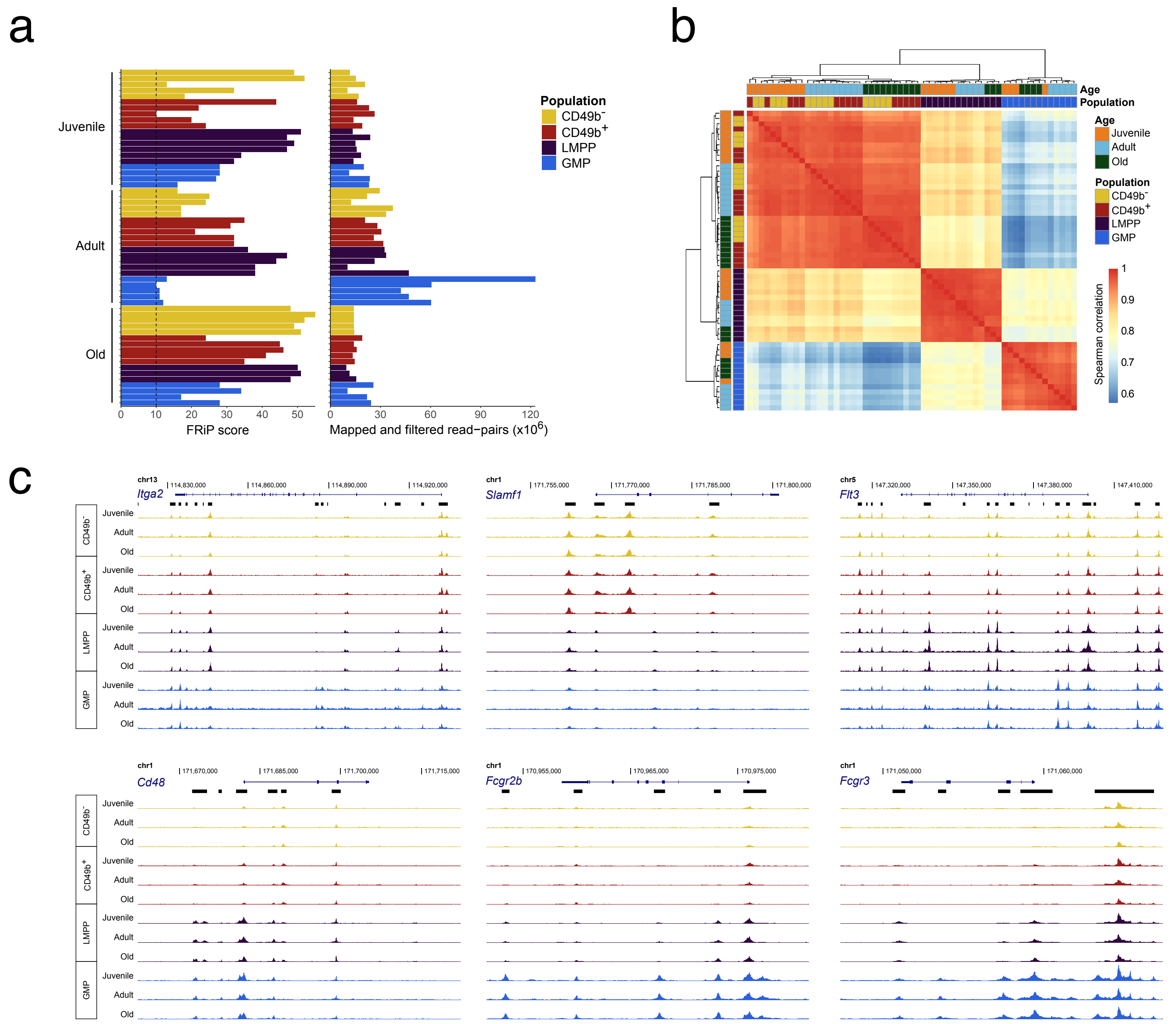

Supplementary Fig. 7, related to Figure 5

ATAC-seq sample characteristics and chromatin accessibility of selected regions. a, Distribution of fraction of reads in peaks (FRiP) score and number of mapped and filtered read-pairs among ATAC-seq samples. **b,** Spearman correlation between ATAC-seq samples. Correlation is calculated on $\log_{10}$ transformed and quantile normalized data. **c,** UCSC browser tracks of median ATAC-seq signal for selected, characteristic cell surface markers for the studied cell populations.

Source data are provided as a Source Data file.

Supplementary Fig. 8, related to Figure 5

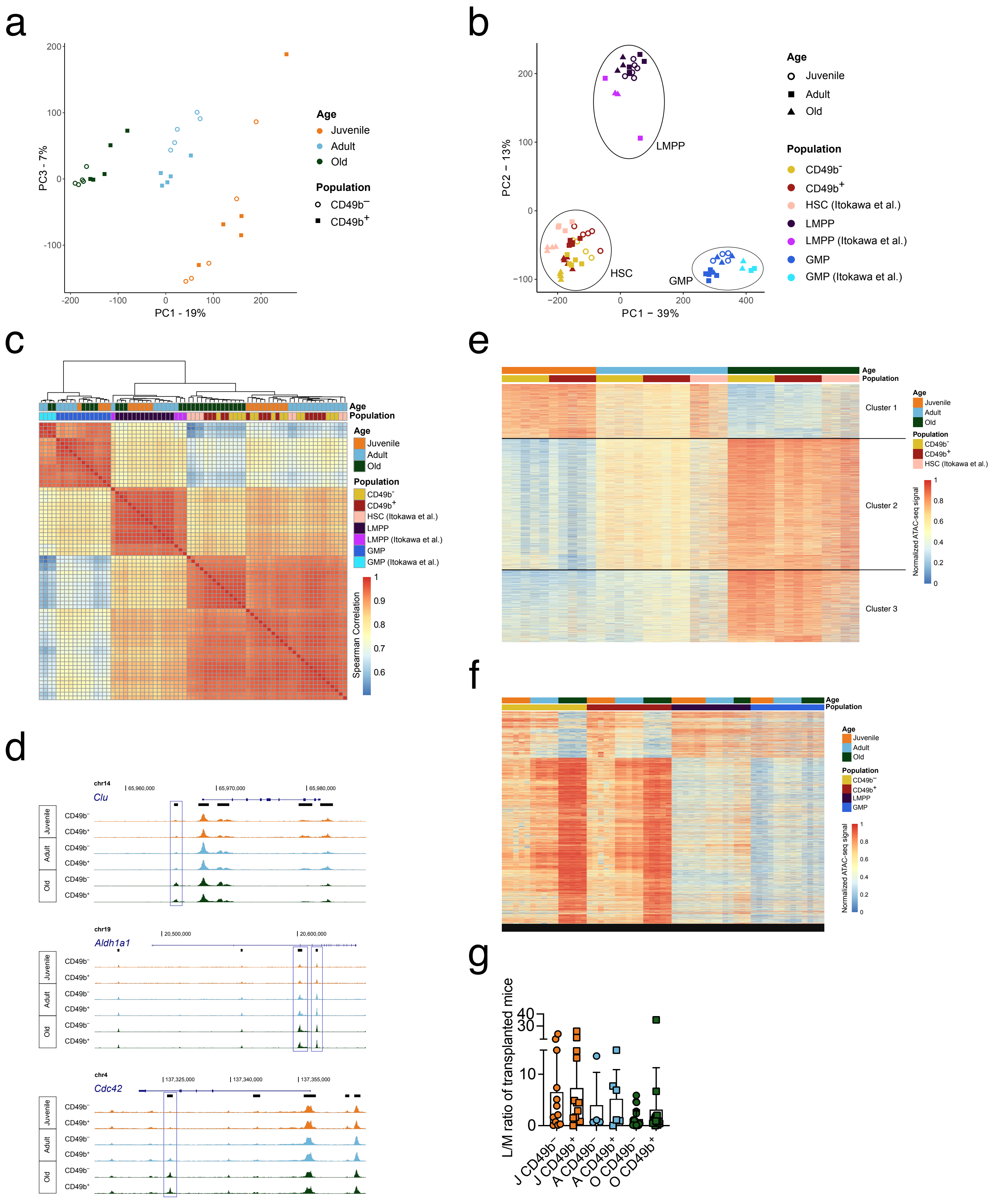

Supplementary Fig. 8, related to Figure 5.

Chromatin accessibility of selected gene regions associated with aging and contextualization of ATAC-seq data in relation to prior studies. a, Principal component analysis of ATAC-seq data from $CD49b^{-}$ and $CD49b^{+}$ HSC subsets, from juvenile, adult, and old mice ($n^{J}_{CD49b^{-}}$=5 mice, $n^{J}_{CD49b^{+}}$=5 mice, $n^{A}_{CD49b^{-}}$=5 mice, $n^{A}_{CD49b^{+}}$=5 mice, $n^{O}_{CD49b^{-}}$=5 mice, $n^{O}_{CD49b^{+}}$=5 mice). Principal components 1 and 3 are shown. **b,** Principal component analysis of ATAC-seq data from stem- and progenitor cells analyzed in this study and corresponding populations from Itokawa et al.[22]. **c,** Hierarchically clustered heatmap of Spearman correlations between samples included in (b). **d,** UCSC browser tracks of median ATAC-seq signal for selected regions. Peaks with significantly changed accessibility between juvenile and old HSCs are indicated with blue boxes. **e,** Heatmap of row normalized chromatin accessibility for regions with differential accessibility (p_{adj}<0.0001, Wald test) between juvenile and old $CD49b^{-}$ and/or between juvenile and old $CD49b^{+}$ cells across HSC samples from this study and Itokawa et al.[22]. **f,** Heatmap of row normalized chromatin accessibility for regions with differential accessibility (p_{adj}<0.0001, Wald test) between juvenile and old $CD49b^{-}$ and/or between juvenile and old $CD49b^{+}$ cells across HSC, LMPP, and GMP samples. **g,** The L/M ratio of PB in mice 5-6 months after transplantation with either the $CD49b^{-}$, or the $CD49b^{+}$ HSC subset from juvenile, adult, and old mice ($n^{J}_{CD49b^{-}}$=14 mice, $n^{J}_{CD49b^{+}}$=24 mice, $n^{A}_{CD49b^{-}}$=5 mice, $n^{A}_{CD49b^{+}}$=10 mice, $n^{O}_{CD49b^{-}}$=20 mice, and $n^{O}_{CD49b^{+}}$=18 mice).

J, juvenile; A, adult; O, old.

Source data are provided as a Source Data file.

Supplementary Fig. 9, related to Figures 5-6

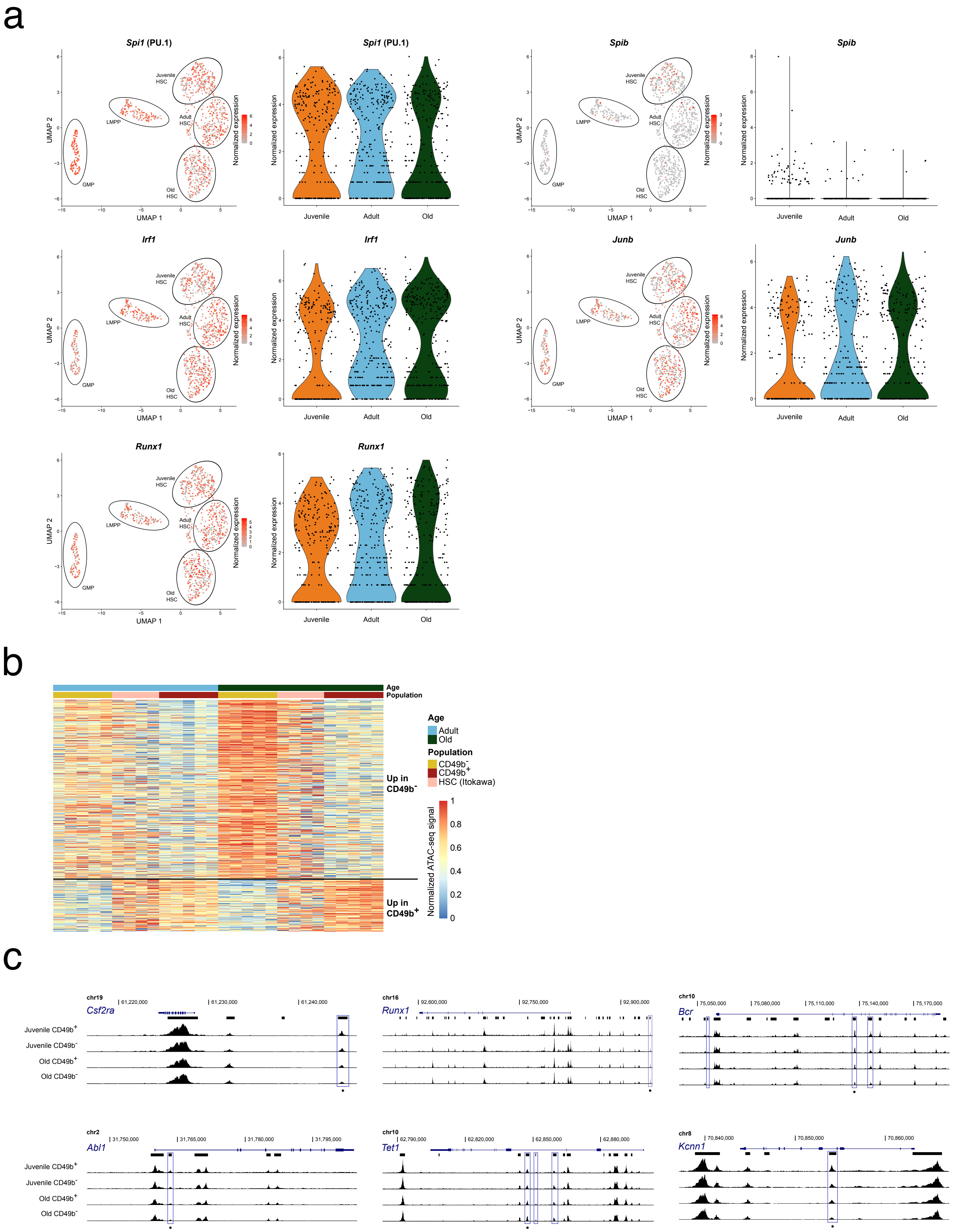

Supplementary Fig. 9, related to Figures 5-6

Gene expression and chromatin accessibility of selected gene regions associated with lineage bias differentiation. **a**, Expressions of *Spi1*, *Spib*, *Irf1, Junb*, and *Runx1* across stem- and progenitor cells from juvenile, adult, and old mice. Normalized expression levels are visualized as color intensity in the UMAP and violin plots for all genes. For *Spib*, the UMAP color scale was cut off at a maximum normalized expression of 3 to improve visibility. **b**, Heatmap of normalized chromatin accessibility for regions with differential accessibility (p_{adj}<0.05, Wald test) between CD49b subpopulations in old mice across HSC samples from this study and Itokawa et al.[22]. **c,** UCSC browser tracks of median ATAC-seq signal for selected regions. Peaks associated with lineage bias differences (Lin DARs) are indicated with blue boxes. Boxed regions indicated with asterisks are shown in Fig. 6f.

Lin DARs, lineage bias associated differentially accessible regions.

Source data are provided as a Source Data file.

Supplementary Table 1. Immunophenotypic definition of hematopoietic cells

**Hematopoietic stem cell compartment**	
CD49b^{-} subset	Lin^{-} Sca-1^{+} c-kit^{+} CD48^{-} CD34^{-} CD150^{hi} CD49b^{-}
CD49b^{+} subset	Lin^{-} Sca-1^{+} c-kit^{+} CD48^{-} CD34^{-} CD150^{hi} CD49b^{+}
CD150^{hi} subset	Lin^{-} Sca-1^{+} c-kit^{+} CD48^{-} CD34^{-} CD150^{hi}
**Bone marrow stem- and progenitor cell compartment**	
Hematopoietic stem cell (HSC)	Lin^{-} Sca-1^{+} c-kit^{+} Flt-3^{-} CD48^{-} CD150^{+} or Lin^{-} Sca-1^{+} c-kit^{+} CD48^{-} CD150^{+}
Common lymphoid progenitor (CLP)	Lin^{-} B220^{low} Sca-1^{low} c-kit^{low} Flt-3^{hi} IL-7Ra^{+}
Lymphoid-primed multipotent progenitor (LMPP)	Lin^{-} Sca-1^{+} c-kit^{+} Flt3^{hi}
Granulocyte-monocyte progenitor (GMP)	Lin^{-} Sca-1^{-} c-kit^{+} CD41^{-} CD150^{-} CD16/32^{+}
Megakaryocyte progenitor (MkP)	Lin^{-} Sca-1^{-} c-kit^{+} CD150^{+} CD41^{+}
**Mature blood lineage cell compartment**	
Platelets	Ter-119^{-} CD41^{+} CD150^{+}
Erythrocytes	CD41^{-} CD150^{-} Ter-119^{+}
Myeloid cells	CD41^{-} Ter-119^{-} CD3e^{-} Thy1.2^{-} NK1.1^{-} CD49b^{-} B220^{-} CD19^{-} CD11b^{+}
B cells	CD41^{-} Ter-119^{-} CD3e^{-} Thy1.2^{-} NK1.1^{-} CD49b^{-} CD11b^{-} Gr-1^{-} B220^{+} CD19^{+}
T cells	CD41^{-} Ter-119^{-} NK1.1^{-} CD49b^{-} CD11b^{-} Gr-1^{-} B220^{-} CD19^{-} CD3e^{+} Thy1.2^{+}
NK cells	CD41^{-} Ter-119^{-} B220^{-} CD19^{-} CD11b^{-} Gr-1^{-} CD3e^{-} Thy1.2^{-} NK1.1^{+} CD49b^{+}
**OP9 B and myeloid lineage differentiation assay**	
Myeloid cells	CD11b^{+}Gr-1^{+} and/or CD11b^{+}F4/80^{+}
B cells	CD19^{+}B220^{+}
**Transmigration and CAFC assay**	
Bone marrow mesenchymal stem cell (BM MSC)	CD45^{-} Ter-119^{-} CD31^{-} CD44^{-} CD51^{+} Sca1^{+}

Supplementary Table 2. Culture conditions for *in vitro* assays

Reagent	Final concentration	Supplier	Catalog number
**OP9 B and myeloid lineage differentiation assay**			
rmSCF	25 ng/ml	Peprotech	250-03
rhFlt3-L	25 ng/ml	Peprotech	300-19
rhIL-7	20 ng/ml	Peprotech	200-07
Penicillin-Streptomycin	1% v/v	Cytiva Hyclone	SV30010
β-mercaptoethanol	0.1mM	Merck	M6250
Fetal bovine serum (FBS)	10% v/v	Cytiva Hyclone	SH30071
Opti-MEM with GlutaMAX		Gibco	51985-026
**Cell division assay**			
rmSCF	50 ng/ml	Peprotech	250-03
rhFlt3-L	50 ng/ml	Peprotech	300-19
rhTpo	50 ng/ml	Peprotech	300-18
rmIL-3	20 ng/ml	Peprotech	213-13
β-mercaptoethanol	0.1mM	Merck	M6250
Fetal bovine serum (FBS)	10% v/v	Cytiva Hyclone	SH30071
x-vivo15 with Gentamicin and L-glutamine		Lonza	BE02-060F
**Megakaryocyte differentiation assay**			
rmSCF	50 ng/ml	Peprotech	250-03
rmFlt3-L	50 ng/ml	Peprotech	300-19
rhTpo	10 ng/ml	Peprotech	300-18
rmIL-3	20 ng/ml	Peprotech	213-13
β-mercaptoethanol	0.1mM	Merck	M6250
Fetal bovine serum (FBS)	10% v/v	Cytiva Hyclone	SH30071
BIT 9500	20% v/v	Stem Cell Technologies	9500
x-vivo15 with Gentamicin and L-glutamine		Lonza	BE02-060F
**BM MSC maintenance assay**			
DMEM with GlutaMAX		Gibco	31966
FBS	10% v/v	Gibco	10500064
HEPES	10 mM	Cytiva Hyclone	SH30237.01
Penicillin-Streptomycin	100 U	Cytiva Hyclone	SV30010
β-mercaptoethanol	0.1mM	Sigma-Aldrich	M7154
**Transmigration and CAFC co-culture assay**			
MyeloCult H5100		Stem Cell Technologies	05150
Hydrocortisone	1 µM	Stem Cell Technologies	74142
Penicillin-Streptomycin	100 U	Cytiva Hyclone	SV30010
**Culture conditions**			
**Assay**	**Conditions**		
MSC expansion	Temperature	37°C	
	CO_2	5%	
	O_2	1%	
HSC/MSC co-culture	Temperature	32–33°C	
	CO_2	5%	
	O_2	20%	
