# Supplementary material for: Aging is associated with functional and molecular changes in distinct hematopoietic stem cell subsets
Source: Nat Commun. 2024 Sep 11;15:7966. doi: 10.1038/s41467-024-52318-1 (PMC11391069; doi:10.1038/s41467-024-52318-1)
Supplement: Supplementary file 3 — Description of additional supplementary files [file 41467_2024_52318_MOESM3_ESM.pdf]

<u>Description of Additional Supplementary Files</u>

Supplementary Data 1: Information about the scRNA-seq dataset.

Supplementary Data 2: Information about the ATAC-seq dataset.

Supplementary Data 3: Antibody list.
