# Supplementary material for: Aging is associated with functional and molecular changes in distinct hematopoietic stem cell subsets
Source: Nat Commun. 2024 Sep 11;15:7966. doi: 10.1038/s41467-024-52318-1 (PMC11391069; doi:10.1038/s41467-024-52318-1)
Supplement: Supplementary file 7 — Reporting Summary [file 41467_2024_52318_MOESM7_ESM.pdf]

nature portfolio

Corresponding author(s): Sidinh Luc

Last updated by author(s): Aug 29, 2024

Reporting Summary

Nature Portfolio wishes to improve the reproducibility of the work that we publish. This form provides structure for consistency and transparency in reporting. For further information on Nature Portfolio policies, see our Editorial Policies and the Editorial Policy Checklist.

Statistics

For all statistical analyses, confirm that the following items are present in the figure legend, table legend, main text, or Methods section.

n/a	Confirmed	
☐	☒	The exact sample size (*n*) for each experimental group/condition, given as a discrete number and unit of measurement
☐	☒	A statement on whether measurements were taken from distinct samples or whether the same sample was measured repeatedly
☐	☒	The statistical test(s) used AND whether they are one- or two-sided<br>*Only common tests should be described solely by name; describe more complex techniques in the Methods section.*
☒	☐	A description of all covariates tested
☐	☒	A description of any assumptions or corrections, such as tests of normality and adjustment for multiple comparisons
☐	☒	A full description of the statistical parameters including central tendency (e.g. means) or other basic estimates (e.g. regression coefficient) AND variation (e.g. standard deviation) or associated estimates of uncertainty (e.g. confidence intervals)
☐	☒	For null hypothesis testing, the test statistic (e.g. *F*, *t*, *r*) with confidence intervals, effect sizes, degrees of freedom and *P* value noted<br>*Give P values as exact values whenever suitable.*
☒	☐	For Bayesian analysis, information on the choice of priors and Markov chain Monte Carlo settings
☒	☐	For hierarchical and complex designs, identification of the appropriate level for tests and full reporting of outcomes
☐	☒	Estimates of effect sizes (e.g. Cohen's *d*, Pearson's *r*), indicating how they were calculated

Our web collection on statistics for biologists contains articles on many of the points above.

Software and code

Policy information about availability of computer code

Data collection	No software was used to collect data.
Data analysis	ATAC-seq and scRNA-seq data was analyzed using open source software.<br>Base processing of scRNA-seq data was done using TopHat (v2.1.1) for mapping and SAMtools (v0.1.18) for deduplication. Subsequent analysis was done in R (v.4.2.2) with Seurat (v.4.3.0). Gene set enrichment analysis was done with GSEA (v4.3.2). The SingleR package (v2.0.0) was used to annotate cells based on other scRNA-seq datasets. HSC-scores were calculated using the hscScore tool (https://github.com/fionahamey/hscScore).<br>Initial processing of ATAC-seq data to obtain read counts in peaks was done using the nf-core ATAC-seq pipeline (https://github.com/nf-core/atacseq, v.1.0.0) and HOMER (v.4.11). HOMER was also used for motif enrichment analysis. Further analysis was done in R (v.4.2.2). In particular, the preprocessCore package (v.1.60.1) was used for normalization of data, DESeq2 (v.1.38.2) for differential analysis, rGREAT (v.2.0.2) for gene ontology analysis with GREAT (v.4.0.4) and limma (v3.54.2) for batch correction between datasets. Tracks of median read coverage were created using BEDTools (v2.29.2) and visualized in the UCSC genome browser (https://genome.ucsc.edu).<br>Plots were made in R using the packages ggplot2 (v.3.4.2), VennDiagram (v.1.7.3) and pheatmap (v.1.0.12).<br>The code used for analysis is available on GitHub at: https://github.com/KI-LucLab/Aging_is_associated_with_functional_and_molecular_changes_in_distinct_HSC_subsets

For manuscripts utilizing custom algorithms or software that are central to the research but not yet described in published literature, software must be made available to editors and reviewers. We strongly encourage code deposition in a community repository (e.g. GitHub). See the Nature Portfolio guidelines for submitting code & software for further information.

Data

Policy information about availability of data

All manuscripts must include a data availability statement. This statement should provide the following information, where applicable:

- Accession codes, unique identifiers, or web links for publicly available datasets
- A description of any restrictions on data availability
- For clinical datasets or third party data, please ensure that the statement adheres to our policy

The following data availability statement has been included: Juvenile and old mice ATAC-seq and scRNA-seq data have been deposited in the European Nucleotide Archive (ENA) under the accession code PRJEB55627 (https://www.ebi.ac.uk/ena/browser/view/PRJEB55627). Adult ATAC-seq and scRNA-seq data have been previously deposited in ENA under the accession number PRJEB47791 (https://www.ebi.ac.uk/ena/browser/view/PRJEB47791)23. Source data are provided with this paper.
Information about input data and the code used for bioinformatics analysis can be found under the section: Code availability.

Research involving human participants, their data, or biological material

Policy information about studies with human participants or human data. See also policy information about sex, gender (identity/presentation), and sexual orientation and race, ethnicity and racism.

Reporting on sex and gender	*Use the terms sex (biological attribute) and gender (shaped by social and cultural circumstances) carefully in order to avoid confusing both terms. Indicate if findings apply to only one sex or gender; describe whether sex and gender were considered in study design; whether sex and/or gender was determined based on self-reporting or assigned and methods used. Provide in the source data disaggregated sex and gender data, where this information has been collected, and if consent has been obtained for sharing of individual-level data; provide overall numbers in this Reporting Summary. Please state if this information has not been collected. Report sex- and gender-based analyses where performed, justify reasons for lack of sex- and gender-based analysis.*
Reporting on race, ethnicity, or other socially relevant groupings	*Please specify the socially constructed or socially relevant categorization variable(s) used in your manuscript and explain why they were used. Please note that such variables should not be used as proxies for other socially constructed/relevant variables (for example, race or ethnicity should not be used as a proxy for socioeconomic status). Provide clear definitions of the relevant terms used, how they were provided (by the participants/respondents, the researchers, or third parties), and the method(s) used to classify people into the different categories (e.g. self-report, census or administrative data, social media data, etc.) Please provide details about how you controlled for confounding variables in your analyses.*
Population characteristics	*Describe the covariate-relevant population characteristics of the human research participants (e.g. age, genotypic information, past and current diagnosis and treatment categories). If you filled out the behavioural & social sciences study design questions and have nothing to add here, write "See above."*
Recruitment	*Describe how participants were recruited. Outline any potential self-selection bias or other biases that may be present and how these are likely to impact results.*
Ethics oversight	*Identify the organization(s) that approved the study protocol.*

Note that full information on the approval of the study protocol must also be provided in the manuscript.

Field-specific reporting

Please select the one below that is the best fit for your research. If you are not sure, read the appropriate sections before making your selection.

☒ Life sciences ☐ Behavioural & social sciences ☐ Ecological, evolutionary & environmental sciences

For a reference copy of the document with all sections, see nature.com/documents/nr-reporting-summary-flat.pdf

Life sciences study design

All studies must disclose on these points even when the disclosure is negative.

Sample size	No sample size calculations or statistical methods were performed to predetermine the experimental sample size. At least three biological replicates are included in all experiments, derived from a minimum of 2-3 independent experiments. In transplantation experiments, sample size was determined based on the efficiency of transplantation and the number of mice required to obtain at least 10 mice with long-term (>5-6 months) reconstitution in the peripheral blood.
Data exclusions	In transplantation experiments, mice terminated or found dead before 2 months post-transplantation due to complications related to animal welfare were excluded from the experiment. Mice were considered positively reconstituted with ≥0.1% reconstitution in at least one of the blood lineages at 2 months post-transplantation, which additionally was represented by ≥10 events in the CD45.2+ or platelet eGFP+ gate. Mice that did not meet the positive reconstitution criterion were excluded from the continuing data analysis but were included in the total number of mice that were transplanted to calculate the transplantation success. For further details, see Methods: Calculation of reconstitution and lineage bias.

	In one of the old transplantation experiments, 4 mice were excluded due to mislabeling of the transplantation subset that cannot be resolved; therefore, they were excluded to avoid confusion and mis-representation of data.<br>For ATAC-seq, samples with a fraction of reads in peaks (FRiP) below 10% were excluded due to low technical quality.<br>For scRNA-seq, data for all cells is made available in the European Nucleotide Archive (ENA) but cells with <50´000 or >750´000 reads, >10% mitochondrial reads or >10% ERCC spike-in contribution are excluded from analysis.
Replication	Multiple independent experiments were performed for each figure. All experiments in replication of the outcome were successful.
Randomization	3.3-4.6 weeks old mice (Juvenile), 7-18 weeks old mice (Adult), and 77-117 weeks old mice (Old) from both sexes were used in the experiments. In transplantation experiments, mice were not randomly distributed but rather, the mice used for transplantation were equally distributed in terms of age range.
Blinding	No blinding was performed in the experiments.

Reporting for specific materials, systems and methods

We require information from authors about some types of materials, experimental systems and methods used in many studies. Here, indicate whether each material, system or method listed is relevant to your study. If you are not sure if a list item applies to your research, read the appropriate section before selecting a response.

Materials & experimental systems

n/a	Involved in the study
☐	☒ Antibodies
☐	☒ Eukaryotic cell lines
☒	☐ Palaeontology and archaeology
☐	☒ Animals and other organisms
☒	☐ Clinical data
☒	☐ Dual use research of concern
☒	☐ Plants

Methods

n/a	Involved in the study
☒	☐ ChIP-seq
☐	☒ Flow cytometry
☒	☐ MRI-based neuroimaging

Antibodies

Antibodies used

All antibodies used can be found in Supplementary Data 3, in addition to information below. Antibody information below are organized as follows:
Antibody; Clone; Supplier; Catalog number; Dilution; Lot number(s)

Ms Sca-1 (Ly-6A/E) BV605; D7; BioLegend; 108134; 1/100; B301237, B323829
Ms Sca-1 (Ly-6A/E) BV605; D7; BD Biosciences; 563288; 1/100; 1293224, 1221072, 2101911, 2192172
Ms Sca-1 (Ly-6A/E) BV650; D7; BD Biosciences; 740450; 1/100; 2181286, 4079256
Ms/Pig CD117 (c-kit) APC-eF780; 2B8; eBioscience; 47-1171-82; 1/400; 4337566, 2018834
Ms CD48 AF700; HM48-1; BioLegend; 103426; 1/200; B279067, B313138, B353730, B406423
Ms CD48 APC; HM48-1; BioLegend; 103412; 1/200; B214728, B268813
Ms CD150 (SLAM) BV785; TC15-12F12.2; BioLegend; 115937; 1/100; B256325, B289084
Ms CD150 (SLAM) PE-Cy7; TC15-12F12.2; BioLegend; 115914; 1/100; B294975, B312846, B294975, B318344
Ms CD34 FITC; RAM34; eBioscience; 11-0341-85; 1/25; 2159106, 1953178, 2112197
Ms CD34 AF647; RAM34; BD Biosciences; 560230; 1/25; 0030905, 1270224, 2300737, 3179598
Ms CD49b PE-Cy7; HMα2; BioLegend; 103518; 1/400; B219359, B278410
Ms CD49b AF647; HMα2; BioLegend; 103511; 1/800; B243328, B272171
Ms CD49b BV711; HMα2; BD Biosciences; 740704; 1/800; 9334247, 8186916, 9334244
Ms CD105 (Endoglin) BV650; MJ7/18; BD Biosciences; 740609; 1/100; 0058118, 0058119, 9301421, 9301423, 0058116, 0058117, 2210400, 2210402
Ms CD127 (IL7-Ra) BV711; A7R34; BioLegend; 135035; 1/100; B267736, B303402
Ms CD135 (Flt-3) PE; A2F10; BioLegend; 135306; 1/50; B295809, B281642, B295809
Ms CD135 (Flt-3) BV421; A2F10; BD Biosciences; 562898; 1/100; 9070512, 0064149, 9070512, 1307885, 2129284, 4057027
Ms CD16/32 AF700; 93; eBioscience; 56-0161-82; 1/400; 2011232
Ms CD16/32 BUV737; AB93; BD Biosciences; 751697; 1/400; 2101862
Ms CD16/32 (Fc-block) Purified; 2.4G2; BD Biosciences; 553142; 1/200; 9248075, 9297201, 1293770, 3199215
Ms CD45.1 (Ly5.1) BV421; A20; BD Biosciences; 563983; 1/50; 9345565, 0290340, 1124987, 1263484, 3278376
Ms CD45.1 (Ly5.1) BUV395; A20; BD Biosciences; 565212; 1/50; 9060613, 007682, 9060613, 1187834, 1306367, 2082263, 3152684
Ms CD45.2 (Ly5.2) PE; 104; BioLegend; 109808; 1/50; B361505, B271929, B247809, B361505, B298925, 1055670, 3201722
Ms CD45.2 (Ly5.2) APC-Fire750; 104; BioLegend; 109852; 1/25; B247809
Ms CD11b (Mac-1) BUV395; M1/70; BD Biosciences; 563553; 1/800 8339988
Ms CD11b (Mac-1) BUV737; M1/70; BD Biosciences; 564443; 1/800; 8206860, 3128894, 9198300, 1169714
Ms/Hu CD11b (Mac-1) BV510; M1/70; BioLegend; 101263; 1/800; B305919, B265262
Ms CD3e AF700; 500A2; BD Biosciences; 557984; 1/100; 8291881, 9323061, 1110899
Ms CD3e BV510; 145-2C11; BioLegend; 100353; 1/50; B320540, B320540, B340821
Ms CD3e BV510; 1451-2C11; BD Biosciences; 5630024; 1/50; 2129070, 2060074, 2175242, 3177370
Ms CD3e BUV395; 1451-2C11; BD Biosciences; 563565; 1/50; 1055680

Ms CD4 BUV395; RM4-5; BD Biosciences; 740208; 1/800; 9058752
Ms CD29 PE; HMb1-1; BD Biosciences; 562801; 1/100; 0072137
Ms CD5 BV510; 53-7.3; BioLegend; 100627; 1/100; B292240, B318513
Ms CD5 BV510; 53-7.3; BD Biosciences; 563995; 1/100; 1070010, 1137019, 1137019, 3338288, 4079575, 4136795, 2032090
Ms CD5 BUV395; 53-7.3; BD Biosciences; 740206; 1/50; 9140513, 9157811
Ms CD8a BUV395; 53-6.7; BD Biosciences; 563786; 1/400; 8306672
Ms Thy1.2 BV605; 53-2.1; BioLegend; 140318; 1/800; B307536
Ms CD19 PE-Cy7; 6D5; BioLegend; 115520; 1/200; B290857, B313074, B357251
Ms CD45R/B220; BUV395; RA3-6B2; BD Biosciences; 563793; 1/200; 8269770
Ms/Hu CD45R/B220 PE-Dazzle 594; RA3-6B3; BioLegend; 103258; 1/200; B304889
Ms CD45R/B220 BUV661; RA3-6B2; BD Biosciences; 565077; 1/400; 0170444, 9021680, 1064098, 3054742, 3283599
Ms/Hu CD45R/B220 BV510; RA3-6B2; BioLegend; 103248; 1/100; B313156, B313156, B341172
Ms F4/80 APC; BM8; BioLegend; 123116; 1/400; B268075
Ms Gr-1 BV510; RB6-8C5; BioLegend; 108437; 1/800; B266530
Ms Gr-1 BUV395; RB6-8C5; BD Biosciences; 563849; 1/400; 8172988
Ms Gr-1 (Ly-6G/Ly-6C) PE; RB6-8C5; BD Biosciences; 553128; 1/800; 7285656
Ms NK1.1 BUV395; PK136; BD Biosciences; 564144; 1/100; 0335469, 1152295, 1179026, 1277550, 1347766, 2168841, 2257242
Ms Ter-119 BV510; TER-119; BD Biosciences; 563995; 1/100; 0066126, 1055667, 2032090
Ms Ter-119 BV650; TER-119; BD Biosciences; 747739; 1/100; 9210038, 0058147, 1020195, 0058146, 1020194, 1020197, 1020198, 1020199, 1020202, 1020203, 1277707, 1277710, 1277711, 1277713
Ms Ter-119 BUV395; TER-119; BD Biosciences; 563827; 1/100; 8109696
Ms Ter-119 APC; TER-119; Proteintech; APC-65149; 1/200; 2032090
Ms CD41 BV786; MWReg30; BD Biosciences; 740903; 1/50; 0206949, 0206951, 0206952, 9301430, 0352699, 0352700, 0206950, 9140518, 0352701, 0352702, 0352703, 0352704, 1187641, 2087934, 2087935, 2151673
Ms CD41 PE; eBioMWReg30; BD Biosciences; 12-0411-83; 1/400; 1312369
Ms CD41 BUV661; MWReg30; BD Biosciences; 741531; 1/400; 1221743
Ms CD41 BUV805; MWReg30; BD Biosciences; 741966; 1/400; 1195624, 1250452
Ms CD45 PE/Cyanine5; 30-F11; BioLegend; 103110; 1/400; B340540
Ms Ter-119 PE/Cyanine5; TER-119; BioLegend; 116210; 1/400; B277009
Ms CD31 PE/Cyanine7; IM7; BioLegend; 102418; 1/100; B232909
Ms CD44 APC/Cyanine7; 390; BioLegend; 103028; 1/100; B373765
Ms CD51 PE; RMV-7; BioLegend; 104106; 1/50; B240646
7-AAD; ; BD Biosciences; 559925; 1/200; 301663
DAPI; ; ThermoFisher Scientific; D3571; 1/50000; 1804346
Ki-67 FITC; ; BD Biosciences; 556026; 1/10; 1036970
Ki-67 PE; ; BD Biosciences; 567719; 1/10; 328960
BrdU PE; ; BD Biosciences; 556029; 1/50; 8330650
Ms Ly-6A/E (Sca-1) Pacific Blue; D7; BioLegend; 108120; 1/200; B258720

Validation

All antibodies were validated by the manufacturer for their species specificity and flow cytometry application. Before use, all antibodies were individually titrated to obtain optimal dilutions and tested for antibody signal specificity by inclusion of cells known to lack or express the antigen targeted by the antibody. When applicable, isotype controls were used to ensure the specificity of the antibodies. All experiments included fluorescent-minus-one (FMO) controls.

Eukaryotic cell lines

Policy information about cell lines and Sex and Gender in Research

Cell line source(s)

OP9 stromal cell line was provided by Sten Eirik Jacobsen, Karolinska Institutet.
The cell line is also available at ATCC with a catalog number CRL-2749.

Authentication

OP9 cells have distinct morphology and promote differentiation of hematopoietic stem- and progenitor cells into myeloid and B cells. Authentication procedure involves analysis of the ability of OP9 cells to differentiate lymphoid-primed multipotent progenitor cells (LMPPs) into B cells and myeloid cells at the expected and reported frequencies.

Mycoplasma contamination

All OP9 cells were tested by PCR for mycoplasma contamination before use and were found to be negative in all tests.

Commonly misidentified lines
(See ICLAC register)

No commonly misidentified cell lines were used.

Animals and other research organisms

Policy information about studies involving animals; ARRIVE guidelines recommended for reporting animal research, and Sex and Gender in Research

Laboratory animals

Female and male C57BL/6J mice were used. Juvenile mice 3.3-4.6 weeks old, adult mice between 7-18 weeks old, and old mice between 77-117 weeks old (1.5-2 years) were used. For more information, see Methods: Animals
C57BL/6 CD45.1 congenic strains:
1. B6.SJL-PtprcaPepcb/BoyCrl mouse strain CD45.1 (494)
2. B6.SJL-PtprcaPepcb/BoyJ mouse strain CD45.1 (Jax 002014)
B6.129P2-Tg(Gata1-EGFP)Cn (BAC transgenic) back crossed to C57BL/6 CD45.2
Mice were housed with the following housing conditions: 12/12 hours dark/light cycle, 20°C ambient temperature, 50±5% humidity.

Wild animals	The study did not involve wild animals.
Reporting on sex	Both female and male C57BL/6J mice were used in this study, however, we did not include sex as a variable. This decision was based on the primary objective of our research, which was to investigate the fundamental mechanisms of stem cell function. In our previous studies (DOI: 10.1093/stcltm/szad057), we have not observed any significant sex differences in these mechanisms. Additionally, due to limitations in our sample size, including sex as a variable would not have allowed for a meaningful analysis of sex-specific differences. However, we have reported the sex of the animals for individual experiments in the Source Data file to allow for future analyses if necessary.
Field-collected samples	The study did not involve samples collected from the field.
Ethics oversight	All experiments were approved by the regional ethical committee in Linköping, Sweden (ethical numbers: 882 and 02250-2022), to the Principal Investigator, Sidinh Luc.

Note that full information on the approval of the study protocol must also be provided in the manuscript.

Plants

Seed stocks	*Report on the source of all seed stocks or other plant material used. If applicable, state the seed stock centre and catalogue number. If plant specimens were collected from the field, describe the collection location, date and sampling procedures.*
Novel plant genotypes	*Describe the methods by which all novel plant genotypes were produced. This includes those generated by transgenic approaches, gene editing, chemical/radiation-based mutagenesis and hybridization. For transgenic lines, describe the transformation method, the number of independent lines analyzed and the generation upon which experiments were performed. For gene-edited lines, describe the editor used, the endogenous sequence targeted for editing, the targeting guide RNA sequence (if applicable) and how the editor was applied.*
Authentication	*Describe any authentication procedures for each seed stock used or novel genotype generated. Describe any experiments used to assess the effect of a mutation and, where applicable, how potential secondary effects (e.g. second site T-DNA insertions, mosiacism, off-target gene editing) were examined.*

Flow Cytometry

Plots

Confirm that:

- [x] The axis labels state the marker and fluorochrome used (e.g. CD4-FITC).
- [x] The axis scales are clearly visible. Include numbers along axes only for bottom left plot of group (a 'group' is an analysis of identical markers).
- [ ] All plots are contour plots with outliers or pseudocolor plots.
- [x] A numerical value for number of cells or percentage (with statistics) is provided.

Methodology

Sample preparation	Bone marrow (BM) single cell suspensions were prepared by crushing femurs, tibiae, and iliac crests isolated from the mice into Phosphate-Buffered Saline supplemented with 5% Fetal Bovine Serum and 2mM Ethylenediaminetetraacetic acid. Unfractionated BM cells were counted on the XP-300-Hematology Analyzer and then stained with antibodies against cell surface marker antigens. For further details, see Methods: Preparation of hematopoietic cells.<br><br>For cell cycle and proliferation analyses, cells were fixed and permeabilized post-staining, and were stained with Ki-67 and DAPI or BrdU for flow cytometry analyses. For further details, see Methods: Cell cycle and proliferation assays.
Instrument	The instruments used were XP-300-Hematology Analyzer, FACSymphony™ A5, LSR Fortessa™, and FACSAria™ Fusion.
Software	All experimental data was collected using BD FACSDiva software. Flow cytometry analyses were performed using FlowJo software version 10.9.0.
Cell population abundance	To ensure obtainment of high purity of the populations of interest, test sorts and reanalyses of all populations were performed. Before and after all single cell sorts, validation of single cell deposition was performed using 488-nm fluorescent beads and microscopic evaluation of 1 bead/well, or by colorimetric assay using horseradish peroxidase driven oxidation of the TMB substrate, which causes color change. Single cell sorts were mainly accompanied with index sorting which enabled post-sort analysis of the cell surface antigens of each single cell sorted.<br>For further details, see Methods: Flow Cytometry analysis of hematopoietic cells.
Gating strategy	Mononuclear cells were gated through FSC-A/SSC-A which then were followed with singlet gating with SSC-H/SSC-W and FSC-H/FSC-W. DAPI-negative or 7-AAD-negative cells were used to exclude dead cells. For HSC analyses, Lin-negative gate was used in order to exclude lineage-positive cells. Fluorescent-minus-one controls were included in all analyses as gate-setting controls. When relevant, backgating strategy was used to ensure proper gate-setting for the populations of interest.

In mature blood lineage analyses on both unfractionated bone marrow and peripheral blood, the mature cells were gated based on positive and negative markers.

☒ Tick this box to confirm that a figure exemplifying the gating strategy is provided in the Supplementary Information.
